# Stressful life events as predictors of refugee adolescents’ subjective mental health need

**DOI:** 10.1093/eurpub/ckac129.609

**Published:** 2022-10-25

**Authors:** Y Namer, A Fretian, D Podar, O Razum

**Affiliations:** School of Public Health, Bielefeld University, Bielefeld, Germany

## Abstract

**Background:**

Asylum seeking and refugee (ASR) adolescents fleeing armed conflict have lived through stressful events. Although not all stressful life events are experienced as post-traumatic stress, they may still lead to subjective need for mental health support. In this study, we assessed which stressful events predicted subjective need.

**Methods:**

We collected and analysed cross-sectional data (February 2019-November 2020) from ASR adolescents aged 11-18, coming from Syria, Afghanistan and Iraq (n = 216). Subjective mental health need was measured with the question “Do you think you have emotional difficulties that you need help with?” and stressful life events (SLE) by the SLE Checklist, a self-report screening tool that asks if participants experienced stressful events in three categories: separation from family, witnessing armed conflict, experiencing violence. Binary logistic regression was run to assess the relative contribution of stressful life events to subjective need.

**Results:**

30.1% of participants reported subjective need for mental health support. Most commonly encountered events were witnessing armed conflict (53.2%) and experiencing the death of a loved one (51.9%). Only one stressful event significantly predicted subjective need for mental health support: separation from family or relatives against one's will (e.g., by police or military) [OR = 6.32, 95%CI(1.79,22.31)].

**Conclusions:**

ASR adolescents who have been separated from their family by force report subjective need for mental health support. It is important to supplement diagnostic tools with subjective report of needing mental health care. ASR adolescents who have experienced separation from famil by force should be given spaces to talk about their need for mental health support. Public health interventions could focus on creating or utilising such spaces where mental health support is available. Crucially, bureaucratic and legal burdens that impede speedy family reunification should be reduced.

**Key messages:**

• ASR adolescents who have been separated from their families by force are more likely to report subjective need for mental health support.

• The higher reported need highlights the importance of reducing bureaucratic and legal burdens that impede fast family reunification.

